# How the Brunswikian Lens Model Illustrates the Relationship Between Physiological and Behavioral Signals and Psychological Emotional and Cognitive States

**DOI:** 10.3389/fpsyg.2021.781487

**Published:** 2022-02-02

**Authors:** Judee K. Burgoon, Rebecca Xinran Wang, Xunyu Chen, Tina Saiying Ge, Bradley Dorn

**Affiliations:** ^1^Center for the Management of Information, University of Arizona, Tucson, AZ, United States; ^2^Management Information Systems, University of Arizona, Tucson, AZ, United States

**Keywords:** nonverbal communication, relational communication, dominance, affection, involvement, trust, similarity, nervousness

## Abstract

Social relationships are constructed by and through the relational communication that people exchange. Relational messages are implicit nonverbal and verbal messages that signal how people regard one another and define their interpersonal relationships—equal or unequal, affectionate or hostile, inclusive or exclusive, similar or dissimilar, and so forth. Such signals can be measured automatically by the latest machine learning software tools and combined into meaningful factors that represent the socioemotional expressions that constitute relational messages between people. Relational messages operate continuously on a parallel track with verbal communication, implicitly telling interactants the current state of their relationship and how to interpret the verbal messages being exchanged. We report an investigation that explored how group members signal these implicit messages through multimodal behaviors measured by sensor data and linked to the socioemotional cognitions interpreted as relational messages. By use of a modified Brunswikian lens model, we predicted perceived relational messages of dominance, affection, involvement, composure, similarity and trust from automatically measured kinesic, vocalic and linguistic indicators. The relational messages in turn predicted the veracity of group members. The Brunswikian Lens Model offers a way to connect objective behaviors exhibited by social actors to the emotions and cognitions being perceived by other interactants and linking those perceptions to social outcomes. This method can be used to ascertain what behaviors and/or perceptions are associated with judgments of an actor’s veracity. Computerized measurements of behaviors and perceptions can replace manual measurements, significantly expediting analysis and drilling down to micro-level measurement in a previously unavailable manner.

## Introduction: Relational Communication and the Brunswikian Lens Model

Relational communication forms the architecture through which social relationships are constructed. As expressed by Hawes (1973), “communication functions not only to transmit information but to bind symbol users (p. 15).” Through ubiquitous verbal and nonverbal relational messages, people reciprocally signal the nature of their interpersonal relationships. Implicit signals express how people regard one another and how they gauge the ongoing status of their interpersonal relationships (Guerrero et al., 2017). The signals form non-orthogonal, generic message themes known as *topoi* (Burgoon and Hale, 1984). Drawn from a synthesis of literature and theorizing from multiple social science disciplines, these *topoi* are universal forms of expressions between humans. They represent the fundamental meanings that define how people relate to one another along such dimensions as dominance, affection, involvement, composure, similarity, and trust.

**FIGURE 1:**
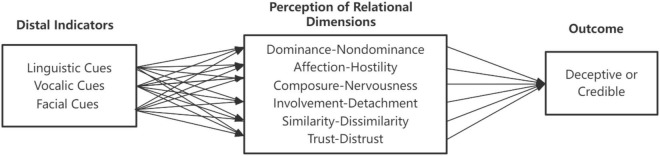
Brunswikian lens model of relational communication.

One way to understand the cognitive and emotional components of relational communication is through the application of a Brunswikian lens model (e.g., Bernieri et al., 1996; Scherer, 2003; Hartwig and Bond, 2011) in which objective *distal indicators* contribute to psychological judgments, also called *proximal percepts*, which are imbued with cognitive or emotional overtones that hold a predictive relationship with outcomes such as deception or credibility. The Brunswikian lens model (Figure 1) brings insight into how relational communication can be expressed either through psychological perceptions or through the kinesic, vocalic and linguistic signals that create those meanings. Some people relate to one another according to the concrete, objective signals, such as “my partner stood seven feet away from me and did not touch me.” Others relate to one another according to the meanings such signals express, such as, “my partner was detached and cold.” These alternative layers of expression can be combined to convey the cognitive and emotional meanings being encoded (expressed) and decoded (deciphered and interpreted). The Brunswikian lens model shows how the different aspects of the signaling process can be combined. The distal, objective signals that can be measured and factored with automated computer tools can be linked to the psychological perceptual judgments that represent relational message themes. These subjective percepts in turn predict communicative outcomes such as successful identification of another’s deception or credibility.

Our demonstration of the lens model comes from a deception project conducted in eight different locations (three in the United States and five in diverse international locations). Groups of 5–8 participants played a game called Resistance, during which they carried out a series of decisions to win (or lose) missions and thus to win (or lose) the game. Those who intended to sabotage the missions employed deception and misdirection, which enabled them to win the game. The interest here is in the automatically measured, objective signals emitted by participants. These formed meaningful clusters that were “read” and responded to as relational messages. We illustrate how a modified Brunswikian lens model combines collections of concrete, objective behaviors to form subjective cognitive and emotional states that represent relational communication. Various relational communication themes in turn predict various social outcomes. Put differently, multimodal distal signals link to proximal percepts of relational messages that, in turn, predict outcomes such as the accurate identification of veracity.

## Methods

### Sample

College-age participants (*N* = 695; mean age = 22 years) from universities in 3 United States states (Arizona, California, and Maryland), and 5 international ones (Israel, Zambia, Fiji, Singapore and Hong Kong) were recruited to participate in an interactive social game called Resistance in exchange for payment for their time and possible bonuses. Universities were ones where local and national IRBs approved participation. The Human Research Protection Office of the United States Army Research Laboratory served as the IRB for the United States institutions and approved the project. The diverse international sample was intended to test the generalizability and universality of findings (see Ting-Toomey et al., 2000, regarding various cultural styles). However, comparisons among the eight locations failed to show significant differences, apart from Fijians expressing more dominance, and sample sizes within United States locations were too small to compare cultural differences, so we have omitted cultural comparisons (see Dunbar et al., 2021; Giles et al., 2021 for the cultural comparisons).

### Procedures

A detailed description of the game is found in Dorn et al. (2021). An ice-breaker activity introducing one another established a baseline for players’ behaviors and perceptions of one another. The games consisted of participants conducting a series of make-believe missions. Teams of up to eight players selected a leader, approved the composition of the teams, then voted for the missions to succeed or fail. Players had been randomly assigned the role of Villager or Spy. Villagers were expected to vote for missions to succeed. Those designated as Spies were expected to engage in occasional deception to cause missions to fail. Spies knew one another’s identity; Villagers did not.

After every other round, players rated other team members on 7-point Likert (1932) format scales measuring each other’s relational communication (see below). The ultimate winners of the game (Spies or Villagers) were determined by which team won the most rounds (see Dorn et al., 2021, for more details). Players also received bonuses if chosen as the leader or a team member.

Nonverbal audiovisual signals (described below) were captured by tablet computers in front of each player, a 360-degree overhead camera and a webcam on the side that recorded the group as a whole. The audiovisual recordings became the basis for kinesic (body language) and vocalic analysis. The audio signals were translated into text for linguistic analysis.

### Affective and Cognitive Measures

The measures that gauged players’ emotional and cognitive states were self-report items from the Relational Communication Scale (RCS; Burgoon and Hale, 1987). These generic themes are context-independent. They represent fundamental dimensions along which people identify how they relate to one another and regard themselves in the context of their interpersonal relationships, without regard to the actual verbal content being expressed. The RCS includes 12 non-orthogonal dimensions, 6 of which were measured here: dominance-nondominance, liking-dislike, involvement-detachment, similarity-dissimilarity, composure-nervousness, and trust-distrust. Coefficient alpha reliabilities were 0.91, 0.89, 0.84, 0.78, 0.84, and 0.91, respectively. Some dimensions that were expected to vary across the time course of the game were measured periodically; others that were expected to be more stable were measured at its conclusion.

### Outcomes/Attributions

Attributions were based on theories of how people relate to one another and use linguistic, kinesic, and vocalic features to express those relationships. Some features appear in multiple relational messages because relational messages are comprised of constellations of nonverbal and verbal signals. For example, lip corner puller that forms smiles appear in liking, composure, involvement, and trust. The typical compositions of these relational message *topoi* can be found in Burgoon et al. (2022).

Table 1 lists the message themes investigated here and the significant linguistic, vocalic and facial features that emerged for each relational dimension. The linguistic features are a small subset of lexical and syntactic features chosen to illustrate their role in conveying relational message themes measured by SPLICE software (Moffitt et al., 2012). The acoustic features are ones that are measured by OpenSmile (Eyben et al., 2010), an open-source software. The facial features are Action Units and combinations measured by the OpenFace software (Baltrušaitis et al., 2015, 2018), also an open-source software program.

**TABLE 1:** Significant linguistic, vocalic, and facial cues of dominance, affection, composure, involvement, similarity, and trust (*p* < 0.1).

Constructs	Linguistic Cues	Vocalic Cues	Facial Cues
Dominance-Non-dominance	Number of Words (+)	Turn-at-talk duration (+) Standard deviation of pitch (+) Average harmonic-to-noise ratio (+) Standard deviation of harmonic-to-noise ratio (−)	Mean cheek raiser (−) Mean lid tightener (+) Mean lip corner puller (+) Variance of brow lowerer (+) Variance of upper lip raiser (+) Variance of dimpler (−) Max inner brow raiser (+) Max outer brow raiser (−) Max brow lowerer (−) Max cheek raiser (+) Max lip corner puller (−) Max dimpler (+)
Affection-Hostility	Number of sentences (+) Hedge ratio (−)	Turn-at-talk duration (+) Average shimmer (−)	Mean cheek raiser (−) Mean dimpler (+) Mean lip tightener (+) Variance of brow lowerer (+) Variance of nose wrinkler (−) Variance of lip tightener (−) Max inner brow raiser (+) Max brow lowerer (−) Max cheek raiser (+) Max lid tightener (−) Max nose wrinkler (+) Max lip corner puller (−)
Composure-Nervousness	Disfluency ratio (−)	Average loudness (+) Average shimmer (−)	Mean upper lip raiser (−) Mean lip stretcher (+) Mean blink (+) Variance of brow lowerer (+) Variance of lip stretcher (−) Max brow lowerer (−) Max nose wrinkler (+) Max chin raiser (−)
Involvement-Detachment	Number of words (+) Number of sentences (+)	Turn-at-talk duration (+) Average shimmer (−)	Mean cheek raiser (−) Mean lid tightener (+) Mean nose wrinkler (+) Mean lip corner puller (+) Variance of brow lowerer (+) Variance of dimpler (−) Max brow lowerer (−) Max cheek raiser (+) Max lid tightener (−) Max dimpler (+)
Similarity-Dissimilarity	Number of sentences (+) Number of words (−)	Standard deviation of harmonic-to-noise ratio (+) Average shimmer (−) Standard deviation of shimmer (+)	Mean inner brow raiser (−) Mean outer brow raiser (+) Mean cheek raiser (−) Mean lip corner puller (+) Mean lip tightener (+) Variance of inner brow raiser (+) Variance of outer brow raiser (−) Variance of brow lowerer (+) Variance of cheek raiser (+) Variance of lip tightener (−) Variance of jaw drop (+) Max lid tightener (−) Max chin raiser (−)
Trust-Distrust	Number of sentences (+)	Turn-at-talk duration (+) Average shimmer (−)	Mean cheek raiser (−) Mean jaw drop (−) Variance of nose wrinkler (−) Variance of jaw drop (+) Max brow lowerer (−) Max lip corner puller (−) Max dimpler (+) Max lip suck (−)

*Positive (and negative) signs in the parentheses indicate significant positive (or negative) unstandardized beta weights in regression analyses between the behavioral cue and the focal relational message construct.*

## Results

Significant indicators are listed in Table 1. Complete statistical results are reported in the Supplementary Material. Here we summarize main findings.

### Dominance-Nondominance

A central theme defining interpersonal relationships is dominance: who is more powerful, who is more subservient, and whether relationships are more egalitarian. In Burgoon and Dunbar (2006), a number of macro-level strategies are outlined for exhibiting power, dominance, and status or their bipolar opposites. In the current analysis we are more concerned with micro-level nonverbal and verbal behaviors through which those strategies are enacted.

As with previous studies (Zhou et al., 2004; Pentland et al., 2021), dominant players talked more often, for a longer duration, and were more likely to contribute to the conversation. Unexpectedly, mean pitch did not correlate with perceptions of dominance. Rather, the standard deviation of pitch had a significant effect on the player’s perceived dominance, indicating dominant individuals talk with more variability in pitch. Further, HNR, which is the proportion of harmonic sound to noise in the voice in decibels (Pentland et al., 2021), was also significant. Higher mean level and lower variability of HNR correlated with a higher perceived dominance. The face was a very active site for signaling dominance or non-dominance. The eye and mouth region were the most involved as dominance signals; language choice played a lesser role.

### Affection-Hostility

Whereas dominance represents the vertical aspect of human relations, affection represents the horizontal dimension. Whether called affiliation, liking, positivity, or valence, this dimension is meant to capture the positive to negative sentiment individuals express toward one another. Many of the behaviors associated with expressions of liking are part of other expressions as well, including expressions of immediacy. Immediacy is an amalgam of proxemic, kinesic, vocalic and linguistic features that signal psychological closeness or distance (Burgoon et al., 1985, 2022). In the case of this game, in which seating location, facing and body orientation, and proxemic behaviors were fixed and therefore excluded from consideration, we looked instead for facial pleasantness, smiling, expressivity and other facial signals of positive affect. Predicted vocalic indicators of liking were pitch variety, relaxed laughter, and rapid turn-switches, while linguistic indicators were predicted to include inclusive language like first person plurals and positive affect language.

Results showed numerous facial expression features correlating with liking and dislike, especially in the mouth, cheek, nose and brow regions. Vocally, only duration of turns-at-talk was positively associated with liking, and mean shimmer (a measure of vocal hoarseness) was negatively associated with liking. Pitch, loudness and other aspects of voice quality did not matter. Longer sentences, less hedging, and (unexpectedly), more dysfluencies were associated with perceived liking.

### Composure-Nervousness

Composure in the case of relational messages means signaling that one is comfortable, at ease and relaxed in the other’s presence. Composure is manifested as facial and postural relaxation. Acoustically, composure presents as a more expressive and pleasant voice. The bipolar opposites of composure are signals of nervousness. In addition to higher anxiety being associated with speech dysfluencies like stuttering (Ezrati-Vinacour and Levin, 2004), nervousness may present in the form of rigid faces, voices, posture and heads; gaze avoidance; fidgeting or other adaptor (self-touching) gestures; softer vocal amplitude; higher pitch; more dysfluencies; and shorter and fewer turns-at-talk. Additionally, nervousness often conveys detachment or unpleasantness (Burgoon et al., 2021).

Results in this experiment showed that more fluent speakers were perceived as more composed, with higher average loudness and lower average shimmer, indicating that those who speak more loudly and less hoarsely are perceived as more composed; conversely, dysfluent, quieter and hoarser voices conveyed discomfort. In terms of facial behaviors, perceived composure (or nervousness) was positively (or negatively) associated with several features in the brow, eye, lip and chin regions, confirming the expectation that nervousness is shown particularly in the upper and lower action units of the face.

### Involvement-Detachment

Involvement is a relational message that can have positive or negative connotations. Dillard et al. (1999) proposed that involvement is an intensifier dimension between competing meanings of dominance or affiliation, which could alter which set of features is associated with involvement. Coker and Burgoon (1987) analyzed over 50 features that could be associated with involvement, most either value-neutral or more tilted in favor of a positive sentiment.

Here, results showed that higher perceived involvement was associated with more words, sentences and longer turns-at-talk duration, indicating that perceived involvement increased with participation in the group conversation. Findings from the audio channel are consistent with Coker and Burgoon (1987), which showed greater involvement corresponded to fewer silences in speech, more vocal warmth and relaxation, but no effect of disfluency. Average magnitude and variability of pitch and loudness were non-significant, contrary to a previous finding that higher pitch, pitch range, and voice intensity are indicative of conversational involvement (Oertel et al., 2011). Meanwhile, perceived involvement was negatively associated with average shimmer. Additionally, significant facial cues included many in the eye, brow and cheek regions. Thus, facial activation played a significant role in expressing involvement.

### Similarity-Dissimilarity

Interpersonal similarity measures the degree to which people share like attitudes, beliefs, personal characteristics, experiences, and so forth (Burgoon and Hale, 1984). Similarity promotes communication and bolsters influence (Krishnan and Hunt, 2021).

The results here showed that, linguistically, the number of sentences was a significant contributor to perceived similarity, while the number of words curiously detracted. Vocally, variability in shimmer had a positive effect on the similarity ratings, while mean shimmer was negatively related. Thus, less overall shimmer but more variability in shimmer expressed similarity. Additionally, perceived similarity was positively associated with the standard deviation of HNR (Harmonic to Noise Ratio), again a signal of variability. It is worth noting that two behavioral indicators, number of sentences and average shimmer, affected the similarity ratings and the trust ratings in the same direction, implying the close relationship between these two relational dimensions. The face model revealed a rich set of significant correlates with similarity, many involving variability or maximums and signifying that more active faces were read as greater similarity.

### Trust-Distrust

As the glue that holds society together, trust plays an essential role in interpersonal (Golembiewski and McConkie, 1975) and commercial (Morgan and Hunt, 1994) relationships and consequently has attracted abundant scholarly attention. Trust fosters cooperation (Balliet and Van Lange, 2013) and reduces costs of social transactions (Dyer and Chu, 2003). Though the concept of trust has been investigated extensively, defining the construct remains a challenging task due to its multi-contextual nature. A typology derived from various definitions (McKnight and Chervany, 2000) suggests that benevolence, integrity, competence, and predictability are the defining characteristics of trust. A rich set of verbal and nonverbal cues, such as smile (Centorrino et al., 2015), eye contact or gaze aversion (Bayliss and Tipper, 2006), facial expressivity (Krumhuber et al., 2007), voice pitch (McAleer et al., 2014), prosody dynamics (Chen et al., 2020), verbal politeness (Lam, 2011) and use of technical terms (Joiner et al., 2002) have been reported to convey interpersonal trust and promote cooperative behavior.

In the current study, we found that the greater number of sentences enhanced a participant’s perceived trustworthiness, though the total amount of speech (i.e., words) had no such effect. The vocalic model showed that turn-at-talk duration, which contributes to the total amount of speech, also boosted perceived trustworthiness, corroborating the positive effect of sentence quantity. Meanwhile, average shimmer had a negative effect on perceived trustworthiness, indicating a less hoarse voice with less breathiness can stimulate trust. The face model produced mixed results. While speaking activity (reflected by the variance of jaw drop) and maximum magnitude of dimpler (a lower face muscle movement driven by smiling) increased perceived trustworthiness, the average level of cheek-raising, jaw-dropping, variance of nose-wrinkling, and maximum level of brow-lowering, lip corner-pulling and lip-sucking all negatively affected trust. Apparently, too much activity and adaptor behavior in the lip and cheek region diminished trust, contrary to the benefit of such vocal and facial activity in expressing involvement and similarity.

### Perceived Veracity

One way to analyze the effect of the six relational dimensions on the outcome of perceived veracity is to use two-stage least squares regression with deception manipulation (i.e., players’ role) as an instrumental variable. We operationalized perceived deceptiveness as the percentage of Villagers who regarded a player as a Spy. Results in the Supplementary Material show that the regression coefficients for all the relational dimensions are significantly negative, suggesting that players with higher perceived dominance, affection, composure, involvement, similarity (with Villager raters), and trustworthiness are less often judged as deceivers. Composure and affection have the largest effect sizes. Thus, players whose relational communication includes nonverbal and verbal signals that convey the least nervousness and engender the most liking are least likely to be suspected as Spies. This analysis demonstrates how the Brunswikian lens model links distal communication signals to meaningful psychological and emotional percepts of interaction to social outcomes of that interaction (e.g., perceived veracity).

## Discussion

Interactants in social contexts send and interpret relational messages using a broad array of verbal and nonverbal behaviors. Applying a modified Brunswikian lens model, we investigated how individuals form proximal percepts based on multimodal behavioral indicators.

We undertook the current approach to illustrate how multimodal signals can be combined to predict some focal variable of interest. Our indicators were not intended to be exhaustive but rather a sampling that could be incorporated into a Brunswikian lens model and thus demonstrate how perceptual and objective variables can be combined to predict whatever outcome is of interest, in this case, deception. Objective distal indicators combine to form proximal percepts; subjective percepts predict outcomes. Modeling social behavior in this manner makes clear the importance of distinguishing objective indicators from subjective perceptions. Distal indicators usually represent more objective, discrete, and microscopic variables that are often regarded as ground truth, whereas percepts are the subjective, macroscopic, interpretive layer of judgments that are formed from the distal cues. Percepts are the intermediate judgment that predicts outcomes of interest. In the case of deception, distal clues might include objective behaviors such as eye blinks and immobile facial muscles that lead to the percept nervousness and thus to the conclusion that the speaker’s frozen, impassive face conveys deceptiveness.

The Brunswikian lens model is a very flexible model that permits choosing few or many indicators of a given type (e.g., facial expressiveness signals), depending on the research question of interest. It also permits beginning with the most distal physical and physiological indicators, then working to the more proximal interior psychological and emotional states to arrive at a predicted behavioral outcome, or instead beginning with the psychological emotional and cognitive states, such as emotional stress and cognitive overload, then working backward to the objective behaviors that account for those cognitive-emotional states. Either the distal indicators or proximal percepts can be used to predict ultimate attributions. Here, where our interest was in deception, the analysis showed that relational messages are one way to conceptualize the implicit social meanings that are the percepts predicting deceptiveness.

Important from a communication (Subrahmanian et al., 2021) standpoint is that all three modalities—linguistic, vocalic and kinesic–contribute variance to the final prediction. The model encourages deeper investigation into what objective indicators contribute to the relational *topoi* that are so deeply embedded in the process of interpersonal communication. An example: A member of a decision-making group may characterize another member’s communication as involved, expressing commonality and similarity, and engendering trust. But these interpretive characterizations leave unanswered what behaviors contribute to those perceptions. AI models can probe what distal signals combine to form these relational messages and lead to perceptions that another is credible or deceptive.

Our findings open up many avenues for future CS research into relational communication. First, the CS community could apply state-of-the-art machine learning methods to predict relational messages. These predictions would facilitate a better understanding of dynamic human interactions. They might show, for instance, how certain actions lead to distrust among group members and account for deterioration of a sense of homophily and liking as the group’s interaction unfolds. Or they might identify what group members’ behaviors promote trust and ultimately, to favorable decisions. Such analysis could assist with decision making scenarios such as business negotiations or discussions of pandemic relief programs. One possible direction is to make inferences on multiple non-orthogonal relational messages through transfer learning (Zhuang et al., 2020). Another direction would be to apply time series analysis to model long interactions, which would allow predictions of dynamic changes in these relational messages over time. Besides making predictions, recent developments in explainable artificial intelligence (Adadi and Berrada, 2018) would help interpret the models and benefit the social science community in identifying more nuanced behavioral indicators of relational messages and in developing relevant theories. Presenting intelligible explanations also increases users’ trust (Gunning et al., 2019). These advancements in CS research present exciting opportunities to further investigate relational messages during human interactions and create synergy between the CS and social science communities.

Second, it would be of great value for the CS community to develop more powerful tools for analyzing behaviors of multiple modalities. Besides the linguistic, vocalic, and facial features, other physiological and behavioral signals, such as gestures and posture, would also be valuable to investigate. In addition, an integrated tool for processing speech, voice, and video in real-time would be beneficial. Although real-time speech (Gao et al., 2019), voice (Acharya et al., 2018), and video processing (Ananthanarayanan et al., 2017) and their integration (Kose and Saraclar, 2021) have been widely studied in computer science, the analysis of physiological and behavioral signals in psychological, emotional, and cognitive states and relational messages presents a new and interesting path, especially for real-time applications (e.g., decision support in business negotiations). Another useful future direction is to harness the power of computer-based techniques to perform real-time audio and video quality checks for better data inputs in a non-laboratory setting. Although we have taken extensive actions to ensure the quality of data collected in labs, unexpected factors, such as uneven lights and background noise, may distort the data collected in the field or in online experiments. A real-time data input quality checker would provide guidance on high-quality data collection and reduce the influence from unforeseen human and environmental matters. We urge further developments in these automated tools for better data collection and analysis.

Although computer scientists and social scientists routinely call for more cross-disciplinary collaboration, such lip service is rarely accompanied by true integration of the work. The Brunswikian lens model offers a productive vehicle for creating that collaboration and integration.

## Data Availability Statement

The datasets presented in this article are not readily available because the project is in progress. Upon completion of the grant, the United States Army Research Office (project sponsor) is committed to making the multimodal, de-identified data available. Contact should be made to the respective investigators for the data of interest. Requests to access the datasets should be directed to VS Subrahmanian, Computer Science, Northwestern University, Evanston, IL, United States.

## Ethics Statement

The studies involving human participants were reviewed and approved by The Army Research Laboratory Human Research Protection Office. The participants provided their written informed consent to participate in this study.

## Author Contributions

JB along with other investigators designed the experiments, planned the data analysis, and wrote a portion of the current manuscript. RW, XC, and TG worked collaboratively to conduct data analysis and wrote one relational theme section. BD conducted much of the data set preparation and initial analysis, wrote software to conduct the game, and traveled internationally to conduct the game. All authors contributed to the article and approved the submitted version.

## Conflict of Interest

JB is a principal in Discern Science International, a for-profit entity that conducts credibility analysis. The remaining authors declare the research was conducted in the absence of any commercial or financial relationships that could be construed as a potential conflict of interest.

## Publisher’s Note

All claims expressed in this article are solely those of the authors and do not necessarily represent those of their affiliated organizations, or those of the publisher, the editors and the reviewers. Any product that may be evaluated in this article, or claim that may be made by its manufacturer, is not guaranteed or endorsed by the publisher.
