# Supplementary material for: How the Brunswikian Lens Model Illustrates the Relationship Between Physiological and Behavioral Signals and Psychological Emotional and Cognitive States
Source: Front Psychol. 2022 Feb 2;12:781487. doi: 10.3389/fpsyg.2021.781487 (PMC8847219; doi:10.3389/fpsyg.2021.781487)
Supplement: Supplementary file 1 [file Table_1.docx]

|  | | | *Supplementary Tables* | | | | | | | | | | | | | | | |
| --- | --- | --- | --- | --- | --- | --- | --- | --- | --- | --- | --- | --- | --- | --- | --- | --- | --- | --- |
|  | | | Table 1A. Regression results of the linguistic cues of perceived dominance, affection, composure, involvement, similarity, and trustworthiness. β and se stand for unstandardized β coefficient and standard error, respectively. | | | | | | | | | | | | | | | |
| *Dependent variable:* | | | *Dominance*  *β (se)* | | *Affection*  *β (se)* | | | *Composure*  *β (se)* | | | *Involvement*  *β (se)* | | | *Similarity*  *β (se)* | | | *Trustworthiness*  *β (se)* | |
| Gender (Male = 1) | | | 0.233 (0.143) | | -0.055 (0.100) | | | 0.011 (0.094) | | | 0.157 (0.115) | | | -0.159 (0.104) | | | -0.024 (0.107) | |
| Game Experience | | | -0.057 (0.133) | | -0.091 (0.093) | | | -0.064 (0.088) | | | -0.051 (0.108) | | | -0.041 (0.097) | | | -0.018 (0.100) | |
| Native English Speaker | | | 0.335^**^ (0.134) | | 0.163^*^ (0.094) | | | 0.147^*^ (0.088) | | | 0.303^***^ (0.109) | | | 0.203^**^ (0.098) | | | -0.027 (0.101) | |
| Dominance Ratio | | | -3.347 (4.982) | | 0.758 (3.481) | | | -3.280 (3.276) | | | -2.474 (4.030) | | | -2.460 (3.625) | | | -3.909 (3.748) | |
| Number of Words | | | 0.001^***^ (0.0003) | | -0.0002 (0.0002) | | | 0.0001 (0.0002) | | | 0.001^***^ (0.0002) | | | -0.0005^**^ (0.0002) | | | -0.0003 (0.0002) | |
| Number of Sentences | | | 0.005 (0.003) | | 0.010^***^ (0.002) | | | 0.002 (0.002) | | | 0.005^**^ (0.003) | | | 0.006^***^ (0.002) | | | 0.008^***^ (0.002) | |
| Hedge Ratio | | | 0.005 (1.710) | | -2.452^**^ (1.195) | | | -1.180 (1.124) | | | 0.606 (1.383) | | | 0.787 (1.244) | | | -0.855 (1.287) | |
| Disfluency Ratio | | | 5.113 (5.635) | | 2.082 (3.937) | | | -10.665^***^ (3.705) | | | 2.060 (4.558) | | | -0.005 (4.100) | | | 6.156 (4.239) | |
| Game Role | | | -0.433^***^ (0.134) | | -0.249^***^ (0.094) | | | -0.222^**^ (0.088) | | | -0.499^***^ (0.108) | | | -0.545^***^ (0.097) | | | -0.894^***^ (0.101) | |
| Constant | | | 3.212^***^ (0.327) | | 4.993^***^ (0.228) | | | 5.155^***^ (0.215) | | | 4.310^***^ (0.264) | | | 4.614^***^ (0.238) | | | 4.789^***^ (0.246) | |
| Observations | | | 287 | | 287 | | | 287 | | | 287 | | | 287 | | | 287 | |
| R^2^ | | | 0.429 | | 0.210 | | | 0.105 | | | 0.408 | | | 0.165 | | | 0.296 | |
| Adjusted R^2^ | | | 0.410 | | 0.185 | | | 0.076 | | | 0.389 | | | 0.138 | | | 0.273 | |
| Residual Std. Error | | | 1.079 (df = 277) | | 0.754 (df = 277) | | | 0.710 (df = 277) | | | 0.873 (df = 277) | | | 0.785 (df = 277) | | | 0.812 (df = 277) | |
| F Statistic | | | 23.127^***^ (df = 9; 277) | | 8.202^***^ (df = 9; 277) | | | 3.621^***^ (df = 9; 277) | | | 21.192^***^ (df = 9; 277) | | | 6.068^***^ (df = 9; 277) | | | 12.924^***^ (df = 9; 277) | |
| *Note:* | ^*^p<0.1; ^**^p<0.05; ^***^p<0.01 | | | | | | | | | | | | | | | | | |
|  | Table 1B. Regression results of the vocalic cues of perceived dominance, affection, composure, involvement, similarity, and trustworthiness. β and se stand for unstandardized β coefficient and standard error, respectively. | | | | | | | | | | | | | | | | | |
| *Dependent variable:* | *Dominance*  *β (se)* | | | *Affection*  *β (se)* | | | *Composure*  *β (se)* | | | *Involvement*  *β (se)* | | *Similarity*  *β (se)* | | | *Trustworthiness*  *β (se)* | | | |
| Gender (Male = 1) | 0.336 (0.345) | | | 0.066 (0.252) | | | -0.295 (0.241) | | | 0.092 (0.267) | | -0.109 (0.244) | | | -0.239 (0.268) | | | |
| Game Experience | -0.219 (0.189) | | | -0.094 (0.138) | | | 0.014 (0.132) | | | -0.050 (0.146) | | -0.067 (0.133) | | | -0.117 (0.146) | | | |
| Native English Speaker | 0.207 (0.193) | | | 0.149 (0.141) | | | 0.050 (0.135) | | | 0.320^**^ (0.149) | | 0.081 (0.136) | | | 0.032 (0.150) | | | |
| TaT Duration | 0.006^***^ (0.001) | | | 0.001^*^ (0.001) | | | 0.001 (0.001) | | | 0.005^***^ (0.001) | | 0.0004 (0.001) | | | 0.001^*^ (0.001) | | | |
| F_0_ Mean | -0.009 (0.006) | | | -0.004 (0.005) | | | -0.003 (0.004) | | | -0.005 (0.005) | | 0.003 (0.004) | | | -0.004 (0.005) | | | |
| F_0_ SD | 0.025^*^ (0.013) | | | 0.011 (0.010) | | | -0.001 (0.009) | | | 0.010 (0.010) | | -0.003 (0.009) | | | -0.002 (0.010) | | | |
| Loudness Mean | -1.511 (2.013) | | | 0.537 (1.472) | | | 3.060^**^ (1.404) | | | 0.460 (1.555) | | -1.867 (1.422) | | | 0.414 (1.561) | | | |
| Loudness SD | 2.168 (3.718) | | | 1.485 (2.718) | | | -3.367 (2.593) | | | -1.584 (2.872) | | 2.201 (2.625) | | | -0.038 (2.882) | | | |
| HNR Mean | 0.010^**^ (0.004) | | | 0.001 (0.003) | | | -0.001 (0.003) | | | 0.004 (0.003) | | -0.005 (0.003) | | | -0.006 (0.003) | | | |
| HNR SD | -0.018^*^ (0.010) | | | -0.009 (0.007) | | | -0.010 (0.007) | | | -0.013 (0.008) | | 0.013^*^ (0.007) | | | 0.010 (0.008) | | | |
| Jitter Mean | -18.271 (23.954) | | | 15.012 (17.512) | | | 17.990 (16.709) | | | -1.232 (18.505) | | 25.733 (16.912) | | | 7.019 (18.569) | | | |
| Jitter SD | 6.787 (13.724) | | | -16.226 (10.034) | | | -13.165 (9.574) | | | -4.925 (10.602) | | -14.176 (9.690) | | | -5.176 (10.639) | | | |
| Shimmer Mean | -7.846 (14.839) | | | -21.668^**^ (10.849) | | | -20.360^*^ (10.351) | | | -22.422^*^ (11.464) | | -30.137^***^ (10.477) | | | -24.007^**^ (11.503) | | | |
| Shimmer SD | -1.221 (14.220) | | | 10.526 (10.396) | | | 15.082 (9.920) | | | 14.925 (10.986) | | 21.511^**^ (10.040) | | | 14.740 (11.023) | | | |
| Game Role | -0.327^*^ (0.177) | | | -0.307^**^ (0.130) | | | -0.333^***^ (0.124) | | | -0.391^***^ (0.137) | | -0.537^***^ (0.125) | | | -0.992^***^ (0.137) | | | |
| Constant | 5.303^***^ (1.310) | | | 6.536^***^ (0.958) | | | 6.213^***^ (0.914) | | | 6.555^***^ (1.012) | | 6.112^***^ (0.925) | | | 6.949^***^ (1.015) | | | |
| Observations | 151 | | | 151 | | | 151 | | | 151 | | 151 | | | 151 | | | |
| R^2^ | 0.432 | | | 0.208 | | | 0.155 | | | 0.448 | | 0.262 | | | 0.377 | | | |
| Adjusted R^2^ | 0.369 | | | 0.120 | | | 0.062 | | | 0.386 | | 0.180 | | | 0.307 | | | |
| Residual Std. Error | 1.031 (df = 135) | | | 0.754 (df = 135) | | | 0.720 (df = 135) | | | 0.797 (df = 135) | | 0.728 (df = 135) | | | 0.800 (df = 135) | | | |
| F Statistic | 6.843^***^ (df = 15; 135) | | | 2.359^***^ (df = 15; 135) | | | 1.656^*^ (df = 15; 135) | | | 7.294^***^ (df = 15; 135) | | 3.202^***^ (df = 15; 135) | | | 5.435^***^ (df = 15; 135) | | | |
| *Note:* | ^*^p<0.1; ^**^p<0.05; ^***^p<0.01 | | | | | | | | | | | | | | | | | |
|  | | Table 1C. Regression results of the facial cues of perceived dominance, affection, composure, involvement, similarity, and trustworthiness. β and se stand for unstandardized β coefficient and standard error, respectively. | | | | | | | | | | | | | | | | |
| *Dependent variable:* | | *Dominance*  *β (se)* | | | | *Affection*  *β (se)* | | | *Composure*  *β (se)* | | | | *Involvement*  *β (se)* | | | *Similarity*  *β (se)* | | *Trustworthiness*  *β (se)* |
| Gender (Male = 1) | | 0.243 (0.164) | | | | -0.074 (0.096) | | | -0.065 (0.094) | | | | 0.100 (0.128) | | | -0.087 (0.099) | | -0.078 (0.111) |
| Game Experience | | -0.236 (0.154) | | | | -0.061 (0.090) | | | -0.157^*^ (0.088) | | | | -0.133 (0.120) | | | -0.181^*^ (0.093) | | -0.133 (0.104) |
| Native English Speaker | | 0.202 (0.152) | | | | 0.017 (0.089) | | | 0.041 (0.086) | | | | 0.166 (0.119) | | | -0.052 (0.092) | | -0.151 (0.102) |
| Mean Inner Brow Raiser | | -0.972 (2.272) | | | | -1.995 (1.327) | | | -0.820 (1.294) | | | | -0.518 (1.777) | | | -2.419^*^ (1.375) | | -1.992 (1.529) |
| Mean Outer Brow Raiser | | -1.488 (4.511) | | | | 2.498 (2.636) | | | 2.273 (2.569) | | | | 0.991 (3.528) | | | 4.915^*^ (2.729) | | 4.176 (3.036) |
| Mean Brow Lowerer | | 0.029 (0.273) | | | | -0.048 (0.159) | | | -0.142 (0.155) | | | | 0.053 (0.213) | | | -0.053 (0.165) | | 0.021 (0.184) |
| Mean Upper Lid Raiser | | -0.572 (6.387) | | | | -2.031 (3.732) | | | -0.028 (3.637) | | | | -4.266 (4.995) | | | -4.212 (3.864) | | -5.788 (4.298) |
| Mean Cheek Raiser | | -1.228^***^ (0.375) | | | | -0.366^*^ (0.219) | | | -0.286 (0.213) | | | | -0.700^**^ (0.293) | | | -0.503^**^ (0.227) | | -0.457^*^ (0.252) |
| Mean Lid Tightener | | 0.433^**^ (0.219) | | | | 0.052 (0.128) | | | -0.094 (0.125) | | | | 0.319^*^ (0.171) | | | 0.203 (0.132) | | 0.026 (0.147) |
| Mean Nose Wrinkler | | 6.002 (4.885) | | | | 3.847 (2.854) | | | 0.239 (2.782) | | | | 6.530^*^ (3.820) | | | 4.342 (2.956) | | 5.063 (3.287) |
| Mean Upper Lip Raiser | | -0.161 (0.311) | | | | -0.208 (0.182) | | | -0.365^**^ (0.177) | | | | -0.263 (0.244) | | | -0.239 (0.188) | | -0.071 (0.210) |
| Mean Lip Corner Puller | | 1.133^**^ (0.447) | | | | 0.308 (0.261) | | | 0.131 (0.255) | | | | 0.644^*^ (0.350) | | | 0.502^*^ (0.271) | | 0.343 (0.301) |
| Mean Dimpler | | 0.546 (0.396) | | | | 0.488^**^ (0.231) | | | 0.370 (0.225) | | | | 0.392 (0.310) | | | 0.145 (0.239) | | 0.079 (0.266) |
| Mean Lip Corner Depressor | | -0.529 (1.431) | | | | -0.774 (0.836) | | | -0.729 (0.815) | | | | 0.031 (1.119) | | | -0.319 (0.866) | | 0.174 (0.963) |
| Mean Chin Raiser | | 0.167 (0.837) | | | | -0.358 (0.489) | | | -0.037 (0.477) | | | | -0.008 (0.655) | | | -0.419 (0.506) | | 0.191 (0.563) |
| Mean Lip stretcher | | 0.562 (3.127) | | | | 0.875 (1.827) | | | 3.410^*^ (1.781) | | | | 0.905 (2.445) | | | 0.843 (1.892) | | 1.366 (2.104) |
| Mean Lip Tightener | | 1.117 (2.493) | | | | 2.651^*^ (1.456) | | | 1.255 (1.419) | | | | 2.103 (1.949) | | | 3.293^**^ (1.508) | | 2.135 (1.677) |
| Mean Lips part | | -0.066 (1.019) | | | | -0.339 (0.595) | | | -0.625 (0.580) | | | | -0.180 (0.797) | | | 0.007 (0.617) | | -0.537 (0.686) |
| Mean Jaw Drop | | -0.248 (1.386) | | | | -0.566 (0.810) | | | 0.412 (0.789) | | | | -0.637 (1.084) | | | -1.207 (0.839) | | -1.935^**^ (0.933) |
| Mean Lip Suck | | 4.510 (6.526) | | | | 2.317 (3.813) | | | 4.043 (3.716) | | | | 5.212 (5.103) | | | -3.916 (3.948) | | 2.144 (4.391) |
| Mean Blink | | 2.417 (2.694) | | | | 1.727 (1.574) | | | 3.040^**^ (1.534) | | | | 1.380 (2.106) | | | 0.988 (1.630) | | -0.042 (1.813) |
| Variance of Inner Brow Raiser | | 0.062 (0.944) | | | | 0.689 (0.551) | | | 0.345 (0.537) | | | | 0.080 (0.738) | | | 1.030^*^ (0.571) | | 0.792 (0.635) |
| Variance of Outer Brow Raiser | | 1.581 (1.841) | | | | -0.547 (1.076) | | | -0.741 (1.049) | | | | -0.266 (1.440) | | | -2.449^**^ (1.114) | | -1.741 (1.239) |
| Variance of Brow Lowerer | | 1.672^**^ (0.848) | | | | 1.041^**^ (0.496) | | | 1.221^**^ (0.483) | | | | 1.238^*^ (0.663) | | | 0.898^*^ (0.513) | | 0.702 (0.571) |
| Variance of Upper Lid Raiser | | 2.107 (3.952) | | | | 1.449 (2.309) | | | 0.732 (2.250) | | | | 3.833 (3.090) | | | 2.639 (2.391) | | 2.699 (2.659) |
| Variance of Cheek Raiser | | -1.123 (0.826) | | | | 0.316 (0.483) | | | 0.330 (0.470) | | | | -0.166 (0.646) | | | 1.071^**^ (0.500) | | 0.529 (0.556) |
| Variance of Lid Tightener | | -0.060 (0.537) | | | | 0.295 (0.314) | | | -0.187 (0.306) | | | | 0.031 (0.420) | | | 0.186 (0.325) | | 0.170 (0.361) |
| Variance of Nose Wrinkler | | -3.344 (3.111) | | | | -3.116^*^ (1.818) | | | -0.961 (1.772) | | | | -3.222 (2.433) | | | -2.044 (1.882) | | -3.552^*^ (2.093) |
| Variance of Upper Lip Raiser | | 0.914^*^ (0.535) | | | | -0.048 (0.313) | | | 0.377 (0.305) | | | | 0.480 (0.418) | | | -0.163 (0.324) | | 0.204 (0.360) |
| Variance of Lip Corner Puller | | -0.376 (0.705) | | | | 0.007 (0.412) | | | -0.024 (0.401) | | | | -0.099 (0.551) | | | -0.517 (0.426) | | -0.159 (0.474) |
| Variance of Dimpler | | -1.366^*^ (0.712) | | | | -0.651 (0.416) | | | -0.335 (0.405) | | | | -0.950^*^ (0.557) | | | -0.053 (0.431) | | -0.350 (0.479) |
| Variance of Lip Corner Depressor | | 0.417 (0.473) | | | | 0.355 (0.276) | | | 0.442 (0.269) | | | | 0.233 (0.370) | | | 0.107 (0.286) | | 0.016 (0.318) |
| Variance of Chin Raiser | | 0.364 (0.512) | | | | 0.437 (0.299) | | | 0.214 (0.292) | | | | 0.208 (0.400) | | | 0.382 (0.310) | | 0.112 (0.344) |
| Variance of Lip stretcher | | -0.329 (1.242) | | | | -0.768 (0.726) | | | -1.309^*^ (0.708) | | | | -0.468 (0.972) | | | -0.530 (0.752) | | -0.888 (0.836) |
| Variance of Lip Tightener | | -0.832 (0.968) | | | | -1.288^**^ (0.565) | | | -0.418 (0.551) | | | | -1.243 (0.757) | | | -1.434^**^ (0.585) | | -1.032 (0.651) |
| Variance of Lips part | | 0.277 (0.528) | | | | 0.467 (0.308) | | | 0.348 (0.301) | | | | 0.104 (0.413) | | | 0.321 (0.319) | | 0.425 (0.355) |
| Variance of Jaw Drop | | 0.141 (0.858) | | | | 0.518 (0.502) | | | -0.559 (0.489) | | | | 0.667 (0.671) | | | 1.151^**^ (0.519) | | 1.415^**^ (0.578) |
| Variance of Lip Suck | | -9.919 (8.987) | | | | -4.857 (5.251) | | | -6.282 (5.118) | | | | -10.700 (7.029) | | | 1.470 (5.437) | | -4.623 (6.048) |
| Variance of Blink | | -0.816 (1.320) | | | | -0.536 (0.771) | | | -1.190 (0.752) | | | | -0.057 (1.033) | | | -0.012 (0.799) | | -0.154 (0.889) |
| Max Inner Brow Raiser | | 0.317^*^ (0.187) | | | | 0.199^*^ (0.110) | | | -0.027 (0.107) | | | | 0.177 (0.147) | | | 0.014 (0.113) | | 0.007 (0.126) |
| Max Outer Brow Raiser | | -0.378^**^ (0.165) | | | | -0.126 (0.096) | | | 0.090 (0.094) | | | | -0.182 (0.129) | | | 0.017 (0.100) | | -0.089 (0.111) |
| Max Brow Lowerer | | -0.446^**^ (0.197) | | | | -0.221^*^ (0.115) | | | -0.226^**^ (0.112) | | | | -0.281^*^ (0.154) | | | -0.186 (0.119) | | -0.240^*^ (0.133) |
| Max Upper Lid Raiser | | 0.204 (0.194) | | | | -0.054 (0.113) | | | -0.080 (0.110) | | | | 0.001 (0.152) | | | 0.035 (0.117) | | 0.138 (0.131) |
| Max Cheek Raiser | | 1.044^***^ (0.347) | | | | 0.448^**^ (0.203) | | | 0.183 (0.197) | | | | 0.676^**^ (0.271) | | | 0.133 (0.210) | | 0.329 (0.233) |
| Max Lid Tightener | | -0.334 (0.237) | | | | -0.240^*^ (0.138) | | | -0.066 (0.135) | | | | -0.343^*^ (0.185) | | | -0.267^*^ (0.143) | | -0.125 (0.159) |
| Max Nose Wrinkler | | 0.010 (0.174) | | | | 0.251^**^ (0.102) | | | 0.213^**^ (0.099) | | | | 0.071 (0.136) | | | 0.103 (0.106) | | 0.171 (0.117) |
| Max Upper Lip Raiser | | -0.197 (0.253) | | | | 0.143 (0.148) | | | 0.061 (0.144) | | | | -0.095 (0.198) | | | 0.148 (0.153) | | 0.018 (0.170) |
| Max Lip Corner Puller | | -0.579^*^ (0.331) | | | | -0.353^*^ (0.193) | | | -0.048 (0.189) | | | | -0.411 (0.259) | | | -0.182 (0.200) | | -0.523^**^ (0.223) |
| Max Dimpler | | 0.711^***^ (0.257) | | | | 0.230 (0.150) | | | 0.083 (0.146) | | | | 0.523^***^ (0.201) | | | 0.082 (0.155) | | 0.408^**^ (0.173) |
| Max Lip Corner Depressor | | -0.028 (0.118) | | | | 0.024 (0.069) | | | 0.027 (0.067) | | | | 0.024 (0.093) | | | -0.027 (0.072) | | 0.044 (0.080) |
| Max Chin Raiser | | -0.119 (0.251) | | | | -0.162 (0.146) | | | -0.289^**^ (0.143) | | | | 0.086 (0.196) | | | -0.266^*^ (0.152) | | -0.142 (0.169) |
| Max Lip stretcher | | 0.050 (0.145) | | | | 0.082 (0.085) | | | -0.098 (0.082) | | | | 0.077 (0.113) | | | 0.071 (0.088) | | 0.008 (0.097) |
| Max Lip Tightener | | 0.216 (0.220) | | | | 0.012 (0.128) | | | 0.122 (0.125) | | | | 0.164 (0.172) | | | 0.098 (0.133) | | -0.005 (0.148) |
| Max Lips part | | 0.374 (0.279) | | | | -0.079 (0.163) | | | -0.157 (0.159) | | | | 0.146 (0.219) | | | 0.164 (0.169) | | 0.132 (0.188) |
| Max Jaw Drop | | 0.075 (0.214) | | | | -0.049 (0.125) | | | 0.157 (0.122) | | | | -0.082 (0.167) | | | -0.090 (0.129) | | -0.104 (0.144) |
| Max Lip Suck | | -1.822 (2.008) | | | | 0.629 (1.173) | | | 0.289 (1.144) | | | | 0.160 (1.570) | | | 0.042 (1.215) | | -2.411^*^ (1.351) |
| Max Blink | | -0.069 (0.208) | | | | 0.122 (0.121) | | | 0.044 (0.118) | | | | -0.133 (0.162) | | | -0.003 (0.126) | | 0.143 (0.140) |
| Game Role | | -0.391^***^ (0.140) | | | | -0.208^**^ (0.082) | | | -0.205^**^ (0.080) | | | | -0.395^***^ (0.109) | | | -0.470^***^ (0.085) | | -0.712^***^ (0.094) |
| Constant | | 1.502 (2.471) | | | | 2.995^**^ (1.444) | | | 3.969^***^ (1.407) | | | | 2.041 (1.933) | | | 4.570^***^ (1.495) | | 6.877^***^ (1.663) |
| Observations | | 400 | | | | 400 | | | 400 | | | | 400 | | | 400 | | 400 |
| R^2^ | | 0.239 | | | | 0.274 | | | 0.240 | | | | 0.249 | | | 0.307 | | 0.300 |
| Adjusted R^2^ | | 0.109 | | | | 0.151 | | | 0.111 | | | | 0.122 | | | 0.189 | | 0.181 |
| Residual Std. Error | | 1.256 (df = 341) | | | | 0.734 (df = 341) | | | 0.715 (df = 341) | | | | 0.982 (df = 341) | | | 0.760 (df = 341) | | 0.845 (df = 341) |
| F Statistic | | 1.843^***^ (df = 58; 341) | | | | 2.223^***^ (df = 58; 341) | | | 1.859^***^ (df = 58; 341) | | | | 1.952^***^ (df = 58; 341) | | | 2.603^***^ (df = 58; 341) | | 2.521^***^ (df = 58; 341) |
| *Note:* | | ^*^p<0.1; ^**^p<0.05; ^***^p<0.01 | | | | | | | | | | | | | | | | |
