# Supplementary material for: How the Brunswikian Lens Model Illustrates the Relationship Between Physiological and Behavioral Signals and Psychological Emotional and Cognitive States
Source: Front Psychol. 2022 Feb 2;12:781487. doi: 10.3389/fpsyg.2021.781487 (PMC8847219; doi:10.3389/fpsyg.2021.781487)
Supplement: Supplementary file 2 [file Table_2.docx]

|  | Table 2A. Results of two-stage least squares regression with players’ role (i.e., deception manipulation) as an instrumental variable. β and se stand for unstandardized β coefficient and standard error, respectively. | | | | | |
| --- | --- | --- | --- | --- | --- | --- |
|  | *Dependent variable: deception judgment* | | | | | |
|  | 1. β (se) | 1. β (se) | 1. β (se) | (4) β (se) | (5) β (se) | (6) β (se) |
| Gender: Male | 0.245** (0.103) | -0.176* (0.091) | 0.042 (0.105) | 0.105 (0.077) | -0.188*** (0.054) | -0.076** (0.032) |
| Age | -0.0001 (0.0001) | -0.0001 (0.0001) | -0.0001 (0.0001) | -0.00001 (0.0001) | -0.00001 (0.00004) | 0.00001 (0.00003) |
| Native English Speaker | 0.158* (0.085) | 0.277** (0.109) | 0.235* (0.129) | 0.203** (0.084) | 0.153*** (0.053) | 0.027 (0.033) |
| Game Experience: Yes | 0.282*** (0.097) | 0.159* (0.089) | 0.117 (0.109) | 0.189** (0.079) | 0.192*** (0.053) | 0.118*** (0.034) |
| Horizontal Collectivism | 0.055 (0.062) | 0.214** (0.095) | 0.269** (0.135) | 0.143** (0.069) | 0.037 (0.038) | -0.00002 (0.024) |
| Horizontal Individualism | 0.117** (0.051) | 0.088* (0.050) | 0.114* (0.068) | 0.084* (0.043) | 0.015 (0.025) | -0.001 (0.017) |
| Vertical Collectivism | -0.061* (0.032) | 0.023 (0.034) | -0.004 (0.041) | -0.026 (0.028) | -0.003 (0.019) | -0.003 (0.013) |
| Vertical Individualism | -0.013 (0.024) | -0.005 (0.026) | -0.045 (0.035) | -0.006 (0.022) | 0.026* (0.015) | 0.011 (0.010) |
| Positive Face | 0.039 (0.046) | -0.050 (0.053) | -0.065 (0.069) | -0.014 (0.043) | -0.058* (0.031) | -0.012 (0.020) |
| Negative Face | -0.114* (0.061) | -0.101 (0.064) | -0.023 (0.070) | -0.062 (0.050) | 0.029 (0.032) | 0.033 (0.022) |
| Dominance | -0.703*** (0.180) |  |  |  |  |  |
| Affection |  | -1.252*** (0.347) |  |  |  |  |
| Composure |  |  | -1.769*** (0.617) |  |  |  |
| Involvement |  |  |  | -0.817*** (0.194) |  |  |
| Similarity |  |  |  |  | -0.679*** (0.109) |  |
| Trust |  |  |  |  |  | -0.427*** (0.047) |
| Constant | 2.798*** (0.686) | 5.563*** (1.453) | 7.427*** (2.462) | 3.567*** (0.797) | 3.099*** (0.471) | 2.105*** (0.231) |
| Observations | 625 | 625 | 625 | 625 | 625 | 625 |
| R^2^ | -5.487 | -6.582 | -11.008 | -4.571 | -1.542 | -0.178 |
| Adjusted R^2^ | -5.603 | -6.718 | -11.223 | -4.671 | -1.588 | -0.199 |
| Residual Std. Error (df = 613) | 0.915 | 0.989 | 1.245 | 0.848 | 0.573 | 0.390 |
| *Note:* | ^*^p<0.1; ^**^p<0.05; ^***^p<0.01 | | | | | |
