# Supplementary material for: How the Brunswikian Lens Model Illustrates the Relationship Between Physiological and Behavioral Signals and Psychological Emotional and Cognitive States
Source: Front Psychol. 2022 Feb 2;12:781487. doi: 10.3389/fpsyg.2021.781487 (PMC8847219; doi:10.3389/fpsyg.2021.781487)
Supplement: Supplementary file 3 [file Data_Sheet_1.docx]

| Constructs | Linguistic Cues | Vocalic Cues | Facial Cues |
| --- | --- | --- | --- |
| Dominance-Nondominance | Number of Words (+) | Turn-at-talk duration (+)  Standard deviation of pitch (+)  Average harmonic-to-noise ratio (+)  Standard deviation of harmonic-to-noise ratio (-) | Mean cheek raiser (-)  Mean lid tightener (+)  Mean lip corner puller (+)  Variance of brow lowerer (+)  Variance of upper lip raiser (+)  Variance of dimpler (-)  Max inner brow raiser (+)  Max outer brow raiser (-)  Max brow lowerer (-)  Max cheek raiser (+)  Max lip corner puller (-)  Max dimpler (+) |
| Affection-Hostility | Number of sentences (+)  Hedge ratio (-) | Turn-at-talk duration (+)  Average shimmer (-) | Mean cheek raiser (-)  Mean dimpler (+)  Mean lip tightener (+)  Variance of brow lowerer (+)  Variance of nose wrinkler (-)  Variance of lip tightener (-)  Max inner brow raiser (+)  Max brow lowerer (-)  Max cheek raiser (+)  Max lid tightener (-)  Max nose wrinkler (+)  Max lip corner puller (-) |
| Composure-Nervousness | Disfluency ratio (-) | Average loudness (+)  Average shimmer (-) | Mean upper lip raiser (-)  Mean lip stretcher (+)  Mean blink (+)  Variance of brow lowerer (+)  Variance of lip stretcher (-)  Max brow lowerer (-)  Max nose wrinkler (+)  Max chin raiser (-) |
| Involvement-Detachment | Number of words (+)  Number of sentences (+) | Turn-at-talk duration (+)  Average shimmer (-) | Mean cheek raiser (-)  Mean lid tightener (+)  Mean nose wrinkler (+)  Mean lip corner puller (+)  Variance of brow lowerer (+)  Variance of dimpler (-)  Max brow lowerer (-)  Max cheek raiser (+)  Max lid tightener (-)  Max dimpler (+) |
| Similarity-Dissimilarity | Number of sentences (+)  Number of words (-) | Standard deviation of harmonic-to-noise ratio (+)  Average shimmer (-)  Standard deviation of shimmer (+) | Mean inner brow raiser (-)  Mean outer brow raiser (+)  Mean cheek raiser (-)  Mean lip corner puller (+)  Mean lip tightener (+)  Variance of inner brow raiser (+)  Variance of outer brow raiser (-)  Variance of brow lowerer (+)  Variance of cheek raiser (+)  Variance of lip tightener (-)  Variance of jaw drop (+)  Max lid tightener (-)  Max chin raiser (-) |
| Trust-Distrust | Number of sentences (+) | Turn-at-talk duration (+)  Average shimmer (-) | Mean cheek raiser (-)  Mean jaw drop (-)  Variance of nose wrinkler (-)  Variance of jaw drop (+)  Max brow lowerer (-)  Max lip corner puller (-)  Max dimpler (+)  Max lip suck (-) |
